# P-780. Ultrasensitive Detection of Urine LAM for All Kinds of Tuberculosis Utilizing Optofluidic Single-Molecule Counting Technology

**DOI:** 10.1093/ofid/ofae631.974

**Published:** 2025-01-29

**Authors:** Cathy Le, Tiffany Truong, Gipshu Dave, Justin Nguyen, Phuong-Uyen Tran, Harisha Ramachandraiah, Niamh Nolan, Renee Tobias, Valerie Brachet, Frank Zaugg, Peter Wagner, Johanna Sandlund

**Affiliations:** Fluxus, Inc., Sunnyvale, California; Fluxus, Inc., Sunnyvale, California; Fluxus, Inc., Sunnyvale, California; Fluxus, Inc., Sunnyvale, California; Fluxus, Inc., Sunnyvale, California; Foundation for New Innovative Diagnostics, Geneva, Geneve, Switzerland; Fluxus, Inc., Sunnyvale, California; Fluxus, Inc., Sunnyvale, California; Fluxus, Inc., Sunnyvale, California; Fluxus, Inc., Sunnyvale, California; Fluxus, Inc., Sunnyvale, California; Fluxus, Inc., Sunnyvale, California

## Abstract

**Background:**

Sputum is an imperfect yet widely used sample type for the diagnosis of tuberculosis (TB). Children and people living with HIV (PLHIV) are often unable to produce sputum, testing with culture and nucleic acid amplification tests is costly and has long turn-around time, and sputum cannot be used for diagnosis of extrapulmonary TB. Lipoarabinomannan (LAM) in urine is an established TB biomarker, but currently available tests have poor sensitivity (limit of detection [LoD] ∼250 pg/mL and as low as 13% clinical sensitivity) and are only approved for use in PLHIV. Fluxus’ single-molecule counting technology combines integrated optics and microfluidics on a single chip-based system and achieves ultrasensitive detection of biomarkers. We have developed a prototype urine LAM assay utilizing the Fluxus technology and evaluated the preliminary analytical and clinical performance.

**Figure:**
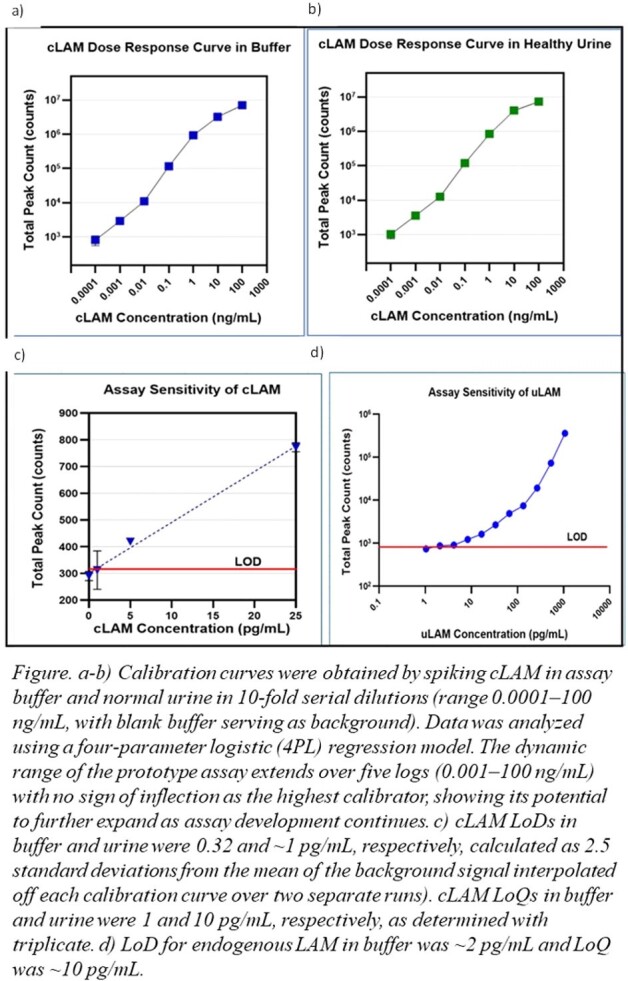

**Methods:**

LoD and dynamic range were determined in buffer and urine by spiking cultured LAM (cLAM) into both matrices. LoD and limit of quantification (LoQ) for endogenous LAM urine (uLAM) were calculated after diluting a urine sample from an individual living with HIV and confirmed TB and with known concentration of uLAM as quantified by MesoScale Diagnostics (MSD) electrochemiluminescence. Dilution linearity and spike recovery activity were measured by spiking cLAM in buffer and normal urine, respectively, in concentrations ranging 0.01–100 ng/mL. Crossreactivity with *Mycobacterium smegmatis* LAM was evaluated with pooled normal urine.

**Results:**

LoDs for cLAM in buffer and urine were 0.32 pg/mL and ∼1 pg/mL, respectively, with a >5-log dynamic range. LoD and LoQ for uLAM were ∼2 and ∼10 pg/mL, respectively. Linearity was acceptable across the range 0.01–100 ng/mL with a mean 102% recovery (range 86%–134%) and mean spike recovery was 106% (range 80%–130%). No crossreactivity with *M. smegmatis* LAM was observed.

**Conclusion:**

Fluxus’ LAM assay, powered by single-molecule counting technology, detects cLAM and uLAM at pg/mL concentrations and with a broad dynamic range. There was minimal matrix effect across the dynamic range of the assay and no crossreactivity to *M. smegmatis* LAM. An ultrasensitive LAM assay has the potential to significantly increase the diagnostic yield and to expand the use of LAM testing to all kinds of TB.

**Disclosures:**

**Cathy Le, BS**, Fluxus, Inc.: Stocks/Bonds (Public Company) **Tiffany Truong, MS**, Fluxus, Inc.: Stocks/Bonds (Public Company) **Gipshu Dave, MS**, Fluxus, Inc.: Stocks/Bonds (Public Company) **Justin Nguyen, BS**, Fluxus, Inc.: Stocks/Bonds (Public Company) **Phuong-Uyen Tran, BS**, Fluxus, Inc.: Stocks/Bonds (Public Company) **Niamh Nolan, MS**, Fluxus, Inc.: Advisor/Consultant **Renee Tobias, MS**, Fluxus, Inc.: Stocks/Bonds (Public Company) **Valerie Brachet, PhD**, Fluxus, Inc.: Stocks/Bonds (Public Company) **Frank Zaugg, PhD**, Fluxus, Inc.: Stocks/Bonds (Public Company) **Peter Wagner, PhD**, Fluxus, Inc.: Stocks/Bonds (Public Company) **Johanna Sandlund, MD, PhD**, Fluxus, Inc.: Stocks/Bonds (Public Company)

